# Oxymatrine inhibits aldosterone-induced rat cardiac fibroblast proliferation and differentiation by attenuating smad-2,-3 and-4 expression: an in vitro study

**DOI:** 10.1186/s12906-016-1231-9

**Published:** 2016-07-26

**Authors:** Lingyun Fu, Yini Xu, Ling Tu, Haifeng Huang, Yanyan Zhang, Yan Chen, Ling Tao, Xiangchun Shen

**Affiliations:** 1 Department of Pharmacology of Materia Medica, Guizhou Medical University, Huaxi University town, Guian New District, Guizhou, 550025 China; 2 The Key Laboratory of Optimal Utilization of Natural Medicinal Resources, Guizhou Medical University, Huaxi University town, Guian new district, Guizhou, 550025 China

**Keywords:** Aldosterone, Oxymatrine, Cardiac fibroblasts, Smad-2, Smad-3, Smad-4

## Abstract

**Background:**

We previously demonstrated oxymatrine, an alkaloid from the Chinese medicine radix *Sophorae flavescentis*, ameliorates hemodynamic disturbances and cardiac fibrosis; however, the underlying mechanisms are unclear. Here, we investigated the effect and mechanism of action of oxymatrine on aldosterone-induced cardiac fibroblast to myofibroblast differentiation in vitro.

**Methods:**

Cardiac fibroblasts were isolated purified from neonatal Sprague Dawley rats. The optimal concentration of aldosterone to stimulate cardiac fibroblast proliferation was determined using the 3-(4,5-dimethylthiazol-2-yl)-2,5-diphenyltetrazolium bromide (MTT) assay. Cardiac fibroblasts were pretreated with 7.57 × 10^−4^ mol/L or 3.78 × 10^−4^ mol/L oxymatrine or without oxymatrine for 2 h, and then coincubated with 1 × 10^−8^ mol/L aldosterone for 48 h. The MTT assay and Masson staining were used to detect the cardiac fibroblast proliferation and myofibroblast differentiation. The secretion of type I and III collagen was measured by commercial ELISA kits, and the hydroxyproline content was determined by the colorimetric assay. Western blotting assayed the Smad-2, Smad-3, and Smad-4 protein expression in cardiac fibroblasts.

**Results:**

The present results confirmed that aldosterone induced cardiac fibroblast to myofibroblast proliferation and differentiation. The MTT assay and Masson staining indicated oxymatrine significantly inhibited aldosterone-induced cardiac fibroblast proliferation and myofibroblast differentiation. Oxymatrine significantly inhibited aldosterone-induced secretion of type I and III collagen, as indicated by commercial ELISA kits, and aldosterone-induced increase in hydroxyproline content, as indicated by a colorimetric assay. Western blotting revealed oxymatrine attenuated aldosterone-induced Smad-2, Smad-3, and Smad-4 expression in cardiac fibroblasts.

**Conclusion:**

Oxymatrine can inhibit cardiac fibroblast proliferation and differentiation into myofibroblasts via a mechanism linked to attenuation of the Smad signaling pathway.

## Background

Cardiovascular diseases are a serious threat to health and are the leading cause of death in humans [1, 2]. The mechanisms leading to cardiovascular diseases and novel drug treatments have undergone intensive research. It is well recognized that cardiac remodeling, the final pathophysiological process of cardiovascular diseases [3], is characterized by three phases: cardiomyocyte hypertrophy and apoptosis, proliferation and differentiation of cardiac fibroblasts, and extracellular matrix deposition. However, most drugs in clinical application aim to prevent cardiomyocyte hypertrophy and apoptosis, including angiotensin converting enzyme inhibitors, beta-receptor blockers and calcium antagonists. In fact, the key pathological changes during cardiac remodeling involve cardiac fibroblasts (CFs), especially CF differentiation into myofibroblasts. The differentiation of CFs results in increased secretion and deposition of myocardial collagen, which induces myocardial stiffness and myocardial diastolic and systolic dysfunction [4–6].

CFs are the main effector cells of cardiac remodeling and can proliferate and differentiate into myofibroblasts, and secrete extracellular matrix proteins such as type I and III collagen [7, 8]. The pathological process of cardiac remodeling involves a variety of factors, including the rennin-angiotensin-aldosterone system (RAAS), growth factor, transforming growth factor-β (TGF-β), and nitric oxide, among others [9]. Accumulating evidence indicates that TGF-β is the one of the key factors that promotes CF differentiation, as direct blockade of TGF-β expression decreases extracellular matrix deposition and tissue fibrosis [10, 11]. Increased expression of TGF-β_1_ and Smad2/3-Smad4 are positively associated with deterioration of cardiac function after myocardial infarction [12, 13]. The levels of CF-secreted endothelin and TGF-β_1_ increase in cells treated with aldosterone (ALD) [14].

**Fig. 1:**
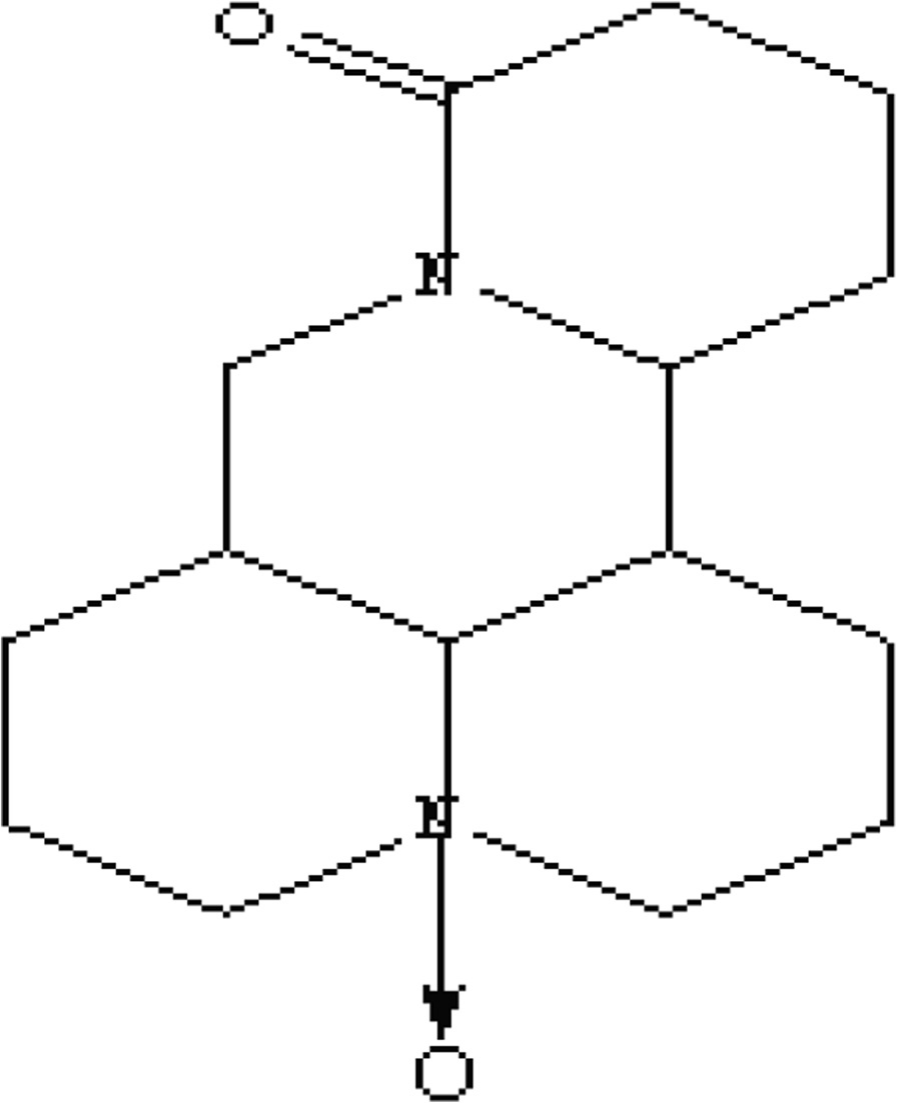
Chemical structure of oxymatrine (OMT)

Oxymatrine (OMT) is one of the main bioactive ingredients of Kushen (*Sophorae flavescentis* radix) which is a traditional Chinese herbal medicine made from the dried root of *S. flavescens* Ait. (Fig. 1). Previous data demonstrated that OMT exerts a wide-range of pharmacological activities, such as anti-inflammatory, anticancer, antiviral and immune regulation effects, and it has been used to treat cardiovascular diseases [15]. Our previous study showed that OMT exerted an inhibitory effect in an experimental model of myocardial fibrosis in rats induced by acute myocardial infarction, via a mechanism involving the TGF-β-Smad signaling pathway [16]. Zhang et al. recently reported that OMT could ameliorate left ventricle hypertrophy and dysfunction in rats with heart failure [17]. Xiao and colleagues reported that OMT exerted a protective effect against ALD-mediated cardiomyocyte injury [18]. Those evidences indicate that OMT can protect the myocardium from apoptosis and fibrosis caused by a variety of stimuli. Hence, the aim of the present study was to further explore the ability of OMT to ameliorate ALD-induced apoptosis in CFs. To the best of our knowledge, this is the first demonstration that OMT protects against ALD-mediated differentiation of CFs to myofibroblasts.

## Methods

### Ethics statement

All animal experiments conformed to the Guide for the Care and Use of Laboratory Animals published by Guizhou Medical University and were approved by the Bioethics Committee for Animal Studies of Guizhou Medical University.

### Materials

OMT (purity, 98 %) was purchased from Green Valley Pharmaceutical Co. Ltd., Shanghai, China; ALD (purity, 98 %) was from Fluka, Switzerland; Trypsin was from Solarbio, Beijing, China; Dulbecco’s modified Eagle’s medium (DMEM) was from GIBCO, Gaithersburg, USA; Penicillin and streptomycin were from Sigma, St. Louis, MO, USA; ELISA assay kits were from Dize Bioengineering, Shanghai; Hydroxyproline assay kits were obtained from Jiancheng Bioengineering, Nanjing, China; and Smad-2,-3 and-4 antibodies were from Cell Signaling Technology, Beverly, USA.

### Isolation and culture of primary neonatal rat CFs

CFs were isolated and purified from 1- to 3-day-old Sprague–Dawley rats. Briefly, the hearts of 1–3 day-old Sprague Dawley rats were isolated and digested in 10 mL of phosphate buffered saline (PBS; 137 mM NaCl, 2.7 mM KCl, 10 mM Na_2_HPO_4_, 2 mM KH_2_PO_4_, pH 7.2-7.4) containing 0.08 % trypsin for 10 min at 37 °C. After each digestion step, the medium containing suspended cells was removed and an equal volume of Spinner/collagenase solution was added. Primary cultures of rat cardiac stromal cells were grown in DMEM supplemented with 20 % fetal bovine serum, penicillin (100 U/mL) and streptomycin (100 U/mL) at 37 °C in a humidified atmosphere of 5 % CO_2_. CFs at the third or fourth passage was used for experiments. The seeding density was 1 × 10^5^ cells/mL for the MTT assay and morphological analyses and 2 × 10^5^ cells/mL for Western blot analysis. The purity of the neonatal rat CF cultures was about 99 %, as indicated by vimentin immunocytochemical staining.

### CF proliferation assay

CFs cultured in 96-well plates were exposed to ALD (1 × 10^−8^ M) alone for 48 h or pretreated with different concentrations of OMT (3.78 × 10^−4^ M to 7.57 × 10^−4^ M) for 2 h before exposure to ALD for 48 h. Then, 3-(4,5-dimethyl-thiazol-2-yl)-2,5-diphenyltetrazolium bromide (MTT) was added to each well (final concentration 0.5 mg/mL) in sterile conditions, and the plates were incubated for 4 h at 37 °C in a 5 % CO_2_ incubator, finally the medium was discarded and washed 3 times with PBS. Formazan salt crystals were dissolved by addition of 150 μL dimethylsulfoxide per well and the absorbance values were determined at 490 nm using a microplate reader (ELX800; GE, USA).

### Enzyme-linked immunosorbent assay (ELISA)

The levels of type I and III collagen in the cell lysis buffer and cell supernatants were measured using ELISA assay kits. The OD values were measured at 450 nm using an ELX800 microplate reader.

### Hydroxyproline colorimetric assay

The hydroxyproline (Hyp) content of the cell lysis buffer and cell supernatants was quantified using a commercial Hyp detection kit. The OD values of the samples were measured at 550 nm using an ELX800 microplate reader.

### Western blotting

Western blotting assays were used to measure the protein expression levels of Smad-2,-3,-4, and β-actin in CFs. After treatment, CFs were washed once in ice-cold PBS, and then lysed in lysis buffer (Dingguo, Beijing, China) on ice. Protein concentrations were assessed using a bicinchoninic acid protein assay kit (Dingguo, Beijing, China). Equal amounts of protein were subjected to 12 % SDS-polyacrylamide gel electrophoresis, transferred onto PVDF membranes using a Bio-Rad Western blot analysis apparatus, and then the membranes were blocked in 5 % non-fat dry milk in TBST, then incubated with primary Smad-2,-3,-4 (1:1000 dilution), and β-actin (1:1000; Cell Signaling Technology) antibodies overnight at 4 °C. After washing three times with TBST, the membranes were incubated with the corresponding secondary antibodies (1:4000, Sigma, MS, USA) for 2 h at room temperature, and the immunolabeled bands were visualized using Pierce ECL Western blotting substrate (Millipore, Bedford, USA).

### Statistical analysis

All data are presented as the mean ± SEM. Between-group comparisons were performed using *t*-tests. All data analysis was performed using Microsoft Excel. Statistical significance was defined as *P* < 0.05; *P* < 0.01 was considered highly significant.

## Results

### OMT inhibits ALD-induced CF proliferation and differentiation

**Fig. 2:**
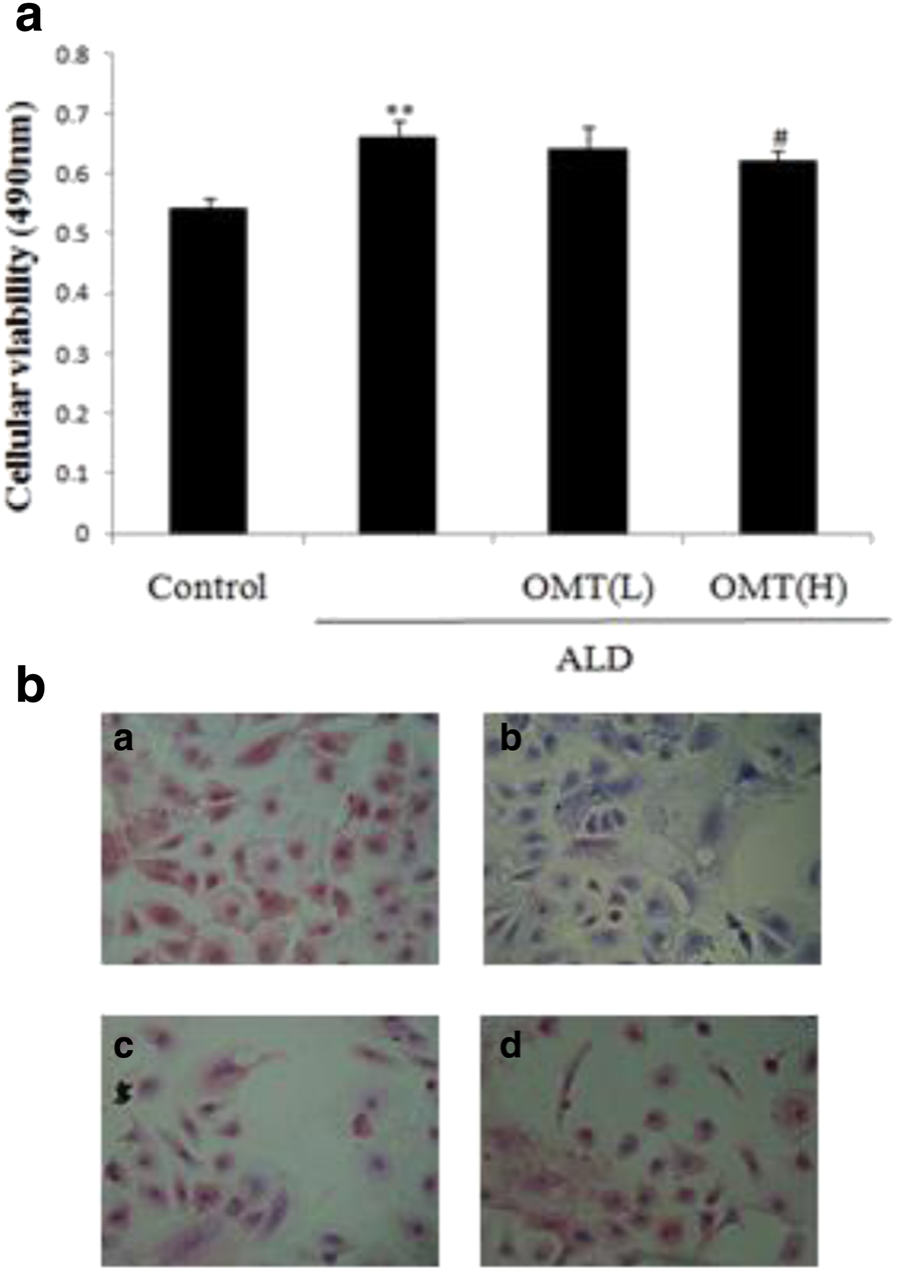
Inhibitory effect of OMT on ALD-induced cardiac fibroblast proliferation. Cardiac fibroblasts were pretreated with 7.57 × 10^−4^ mol/L [High] or 3.78 × 10^−4^ mol/L [Low] OMT or without OMT for 2 h, and then coincubated with ALD (1 × 10^−8^ mol/L) for 46 h. **a** Cell viability was determined using the MTT assay. **b** Collagen fibers were visualized using Masson staining. Representative images of collagen fibers (green or blue), myocardial fibers (*red*), erythrocytes (*orange*), and nuclei (*blue*): (i) Control; (ii) ALD; (iii) OMT (*Low*); (iv) OMT (*High*). Results are means ± SEM of three independent experiments (*P < 0.05, **P < 0.01 vs. control group; ^#^ *P* < 0.05, ^##^ *P* < 0.01 vs. ALD alone, *n* = 5)

The MTT assay and Masson staining were used to assess the ability of OMT to inhibit ALD-induced CF proliferation and differentiation. CFs were pretreated with or without different concentrations of OMT (3.78 × 10^−4^ M and 7.57 × 10^−4^ M) for 2 h and then stimulated with ALD (1 × 10^−8^ M) for 24 h. The MTT assay showed that ALD significantly increased CF proliferation compared to control cells. However, pretreatment with OMT inhibited the ALD-induced increase in CF proliferation (Fig. 2a). CF differentiation was also investigated using Masson staining (Fig. 2b). ALD increased CFs differentiation to myofibroblasts and collagen deposition compared to control cells; however, OMT significantly ameliorated ALD-induced CF differentiation and collagen deposition.

### OMT attenuates ALD-induced type collagen I and collagen III deposition in CFs

**Fig. 3:**
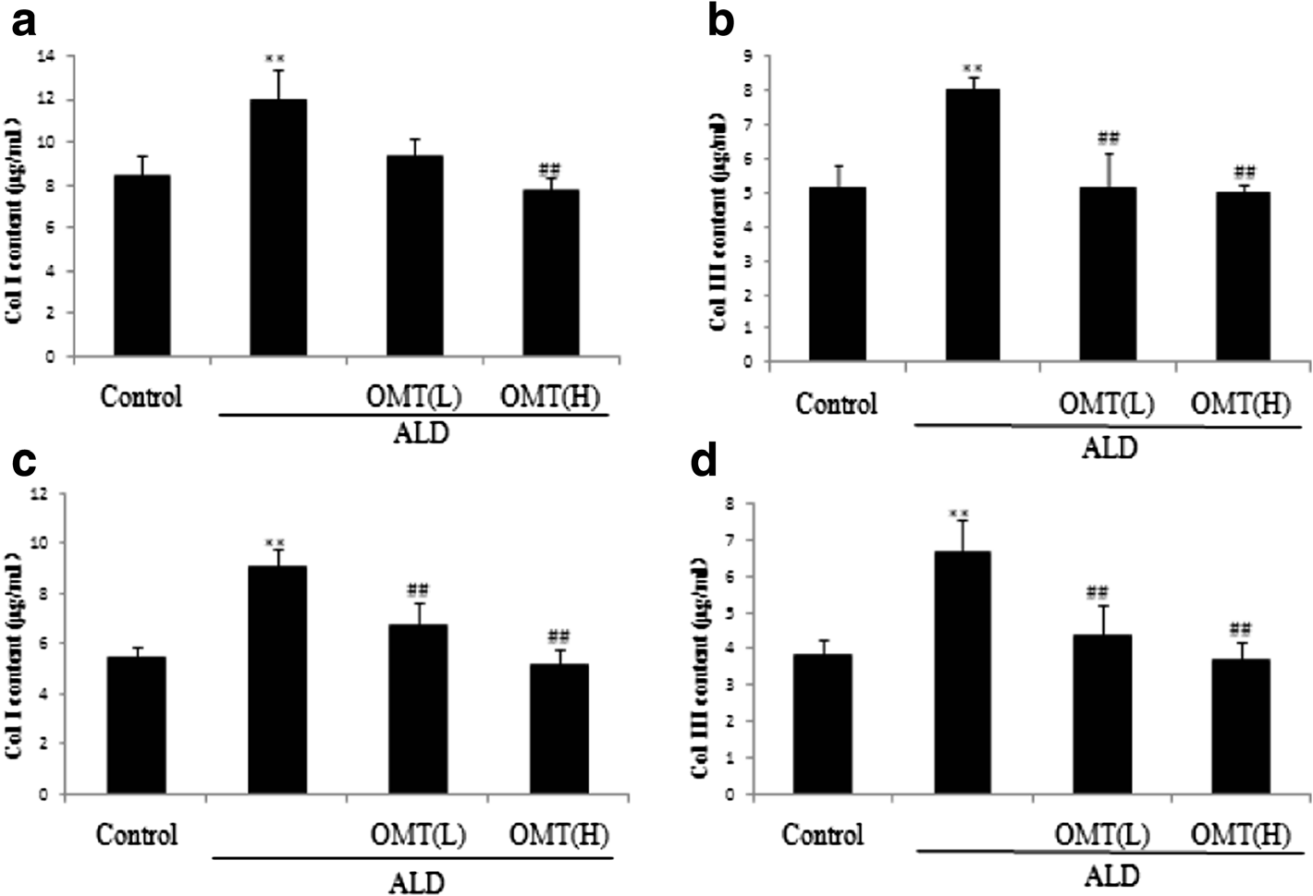
Inhibitory effects of OMT on ALD-induced type I and III collagen secretion of cardiac fibroblasts. Cardiac fibroblasts were pre-treated with 7.57 × 10^−4^ mol/L [High] or 3.78 × 10^−4^ mol/L [Low] OMT or without OMT for 2 h, and then coincubated with ALD (1 × 10^−8^ mol/L) for 46 h. **a** ELISA assays of type I collagen content in cell lysis buffer; (**b**) type III collagen content in cell lysis buffer; (**c**) type I collagen content in cell supernatant; and (**d**) type III collagen content in cell supernatant. Results are means ± SEM of three independent experiments. (*P < 0.05, **P < 0.01 vs. control cells; ^#^ *P* < 0.05, ^##^ *P* < 0.01 vs. ALD alone, *n* = 5)

Type I and III collagen are the key biomarkers of CFs differentiation into myofibroblasts. ELISAs were used to assess the secretion of type I and III collagen. Exposure of CFs to ALD significantly increased the levels of type I and III collagen in the cell lysis buffer and cell supernatant compared to control cells (Fig. 3a-d). However, pretreatment with OMT attenuated the ALD-induced increases in the levels of type I and III collagen in the cell lysis buffer and cell supernatant (Fig. 3a-d).

### OMT inhibits ALD-induced hydroxyproline secretion by CFs

**Fig. 4:**
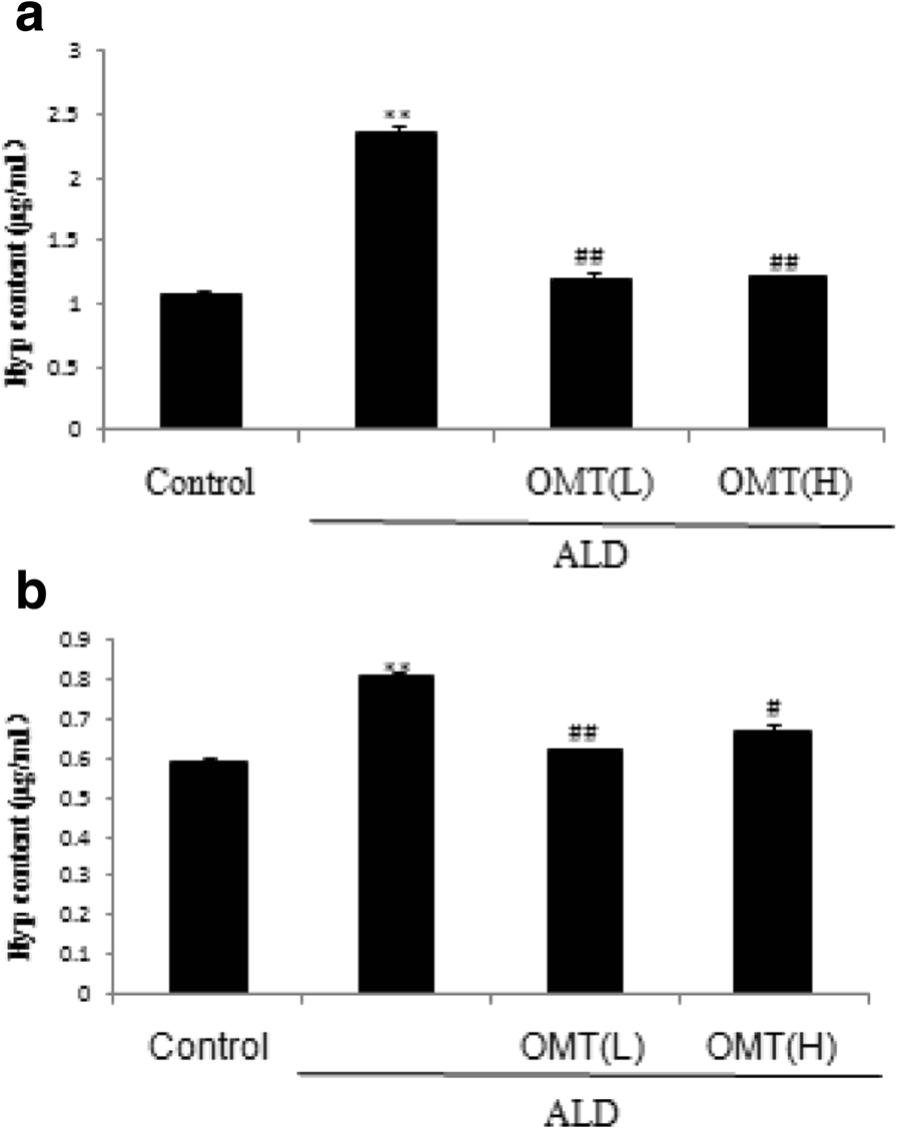
Inhibitory effect of OMT on ALD-induced hydroxyproline (Hyp) secretion by cardiac fibroblasts. Cardiac fibroblasts were pre-treated with 7.57 × 10^−4^ mol/L [High] or 3.78 × 10^−4^ mol/L [Low] OMT or without OMT for 2 h, and then coincubated with ALD (1 × 10^−8^ mol/L) for 46 h. **a** Analysis of hydroxyproline content in cell lysis buffer and (**b**) cell supernatant. Results are means ± SEM of three independent experiments. (*P < 0.05, **P < 0.01 vs. control cells; ^#^ *P* < 0.05, ^##^ *P* < 0.01 vs. ALD alone, *n* = 4)

Hyp, a degradation production of collagen, represents an index of collagen secretion which, to an extent, can reflect myofibroblast secretion activity. Hyp was measured using a commercial colorimetric assay. ALD increased the Hyp content of the cell lysis buffer and cell supernatant of cultured CFs (Fig. 4a-b). Compared to cells treated with ALD, pretreatment with OMT significantly reduced the ALD-induced increase in the Hyp content of the cell lysis buffer and cell supernatant (Fig. 4a-b).

### OMT inhibits ALD-induced expression of smad-2,-3, and-4 in CFs

**Fig. 5:**
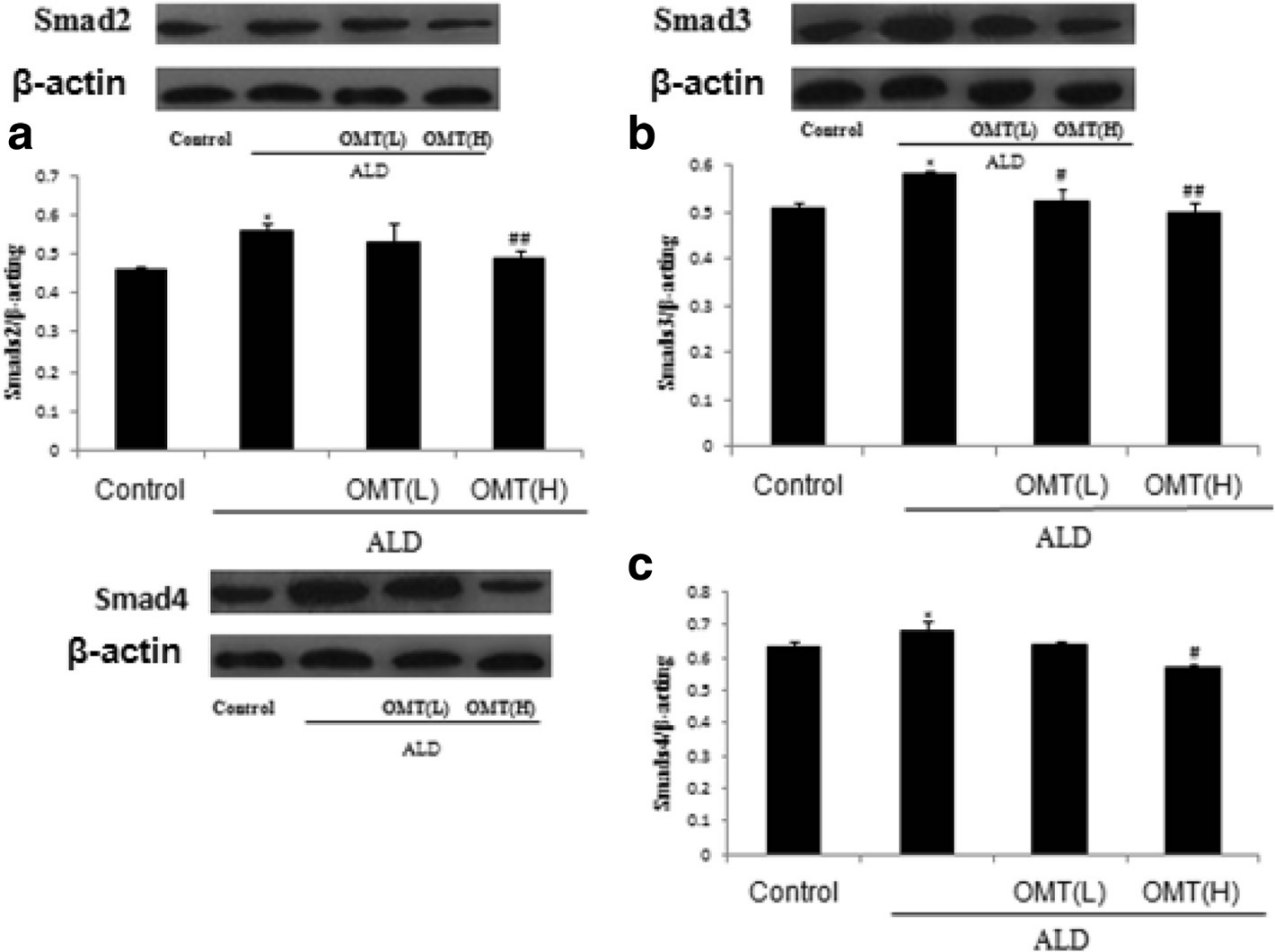
Inhibitory effect of OMT on ALD-induced expression of Smad-2,-3, and-4 in cardiac fibroblasts. Cardiac fibroblasts were pretreated with 7.57 × 10^−4^ mol/L [High] or 3.78 × 10^−4^ mol/L [Low] OMT or without OMT for 2 h, and then coincubated with ALD (1 × 10^−8^ mol/L) for 46 h. Western blotting analysis of (**a**) Smad-2, (**b**) Smad-3 and (**c**) Smad-4. Results are means ± SEM of three independent experiments. (*P < 0.05, **P < 0.01 vs. control cells; ^#^ *P* < 0.05, ^##^ *P* < 0.01 vs. ALD alone, *n* = 4)

The TGF-β-Smad signaling pathway provides the strongest activation signal for organic fibrosis; Smad-2,-3, and-4 are key factors in this signaling pathway. Therefore, we explored Smads protein expression in CFs exposed to ALD. Western blotting showed that ALD enhanced the expression of Smad-2,-3, and-4 in CFs (Fig. 5a-c) compared to control cells. Pretreatment with OMT attenuated the ALD-induced increases in Smad-2,-3, and-4 protein expressions in CFs.

## Discussion

Chronic heart failure is a pathological process caused by cardiac remodeling events, including myocardial hypertrophy, myocardial cell loss, and myocardial fibrosis (MF) [19–21]. Clinical data have confirmed MF occurs as an inevitable process during the progression of heart disease to its terminal stage and the key factors of cardiac function by compensatory period to decompensation period [22, 23]. Therefore, inhibition of abnormal cardiac remodeling, the reaction to target organ damage and fibrosis, by reversing a variety of chronic inflammatory reactions, may represent a primary treatment strategy to improve clinical outcomes and reduce mortality in patients with chronic heart failure [24].

It has been confirmed that the RAAS is a key signal transduction pathway involved in organic fibrosis. Recent reports have indicated that ALD, the final molecule of the RAAS pathway, is widely implicated in myocardial fibrosis and promotes the synthesis of collagen in cardiac CFs [25–28]. In this study, to contribute to the development of novel therapeutic strategies for treating cardiovascular disease, we investigated the ability of OMT to inhibit ALD-induced CF proliferation and differentiation in vitro and explored the associated mechanisms.

The MTT assay showed that CFs exposed to 1 × 10^−8^ mol/L ALD alone for 48 h displayed significantly higher levels of proliferation (*P* < 0.01) compared with control cells. However, pretreatment with OMT significantly attenuated ALD-induced cell proliferation. Masson staining confirmed that OMT significantly reduced ALD-induced collagen fiber accumulation in CFs, and ELISAs suggested that OMT inhibited the ALD-induced secretion of type I collagen, type III collagen and Hyp.

Smad-2,-3, and-4, and the TGF-β1-Smads pathway are implicated in fibrosis. Activation of TGF-β1-Smads is an important signal that leads to cardiac fibrosis. Western blotting showed ALD significantly increased the expression of Smad-2,-3, and-4; these three proteins can promote myocardial fibrosis and play major roles in the TGF-β-Smad signaling pathway. However, OMT significantly inhibited ALD-induced protein expression of Smad-2,-3 and-4 compared to cells treated with ALD alone. These results suggest that OMT may inhibit ALD-induced proliferation and differentiation of CFs via a mechanism linked to the TGF-β/Smad signaling pathway and downregulation of Smad-2,-3 and-4 protein expression. The potential of OMT as a drug to prevent and treat MF merits further research in preclinical models.

## Conclusion

OMT attenuates ALD-induced CF proliferation and differentiation into myofibroblasts via a mechanism that involves the TGF-β-Smad signal transduction pathway. The present study highlights on a novel molecular mechanism by which OMT inhibits ALD-induced CF differentiation into myofibroblasts.

## Abbreviations

ALD, aldosterone; CFs, cardiac fibroblasts; DMEM, dulbecco’s modified Eagle’s medium; Hyp, hydroxyproline; MF, myocardial fibrosis; MTT, 3-(4,5-dimethylthiazol-2-yl)-2,5-diphenyltetrazolium bromide; ; OMT, oxymatrine; PBS, phosphate buffered saline; RAAS, rennin-angiotensin-aldosterone system; TGF-β, transforming growth factor-β
